# The Effect of Leukocyte Removal and Matrix Metalloproteinase Inhibition on Platelet Storage Lesions

**DOI:** 10.3390/cells13060506

**Published:** 2024-03-13

**Authors:** Alina Rak-Pasikowska, Kornela Hałucha, Agnieszka Sapa-Wojciechowska, Aneta Wrzyszcz, Wioletta Gałuszka, Anna Pęcak-Solińska, Iwona Bil-Lula

**Affiliations:** 1 Division of Clinical Chemistry and Laboratory Haematology, Department of Medical Laboratory Diagnostics, Faculty of Pharmacy, Wroclaw Medical University, Borowska 211A St., 50-556 Wrocław, Poland; kornela.halucha@umw.edu.pl (K.H.); agnieszka.sapa-wojciechowska@umw.edu.pl (A.S.-W.); iwona.bil-lula@umw.edu.pl (I.B.-L.); 2 Lower Silesian Oncology, Pulmonology and Hematology Center, 12 Hirszfeld Square, 53-413 Wrocław, Poland; aneta.wrzyszcz@dcopih.pl; 3 Professor Tadeusz Dorobisz Regional Centre for Blood Donation and Haemotherapy in Wrocław, Red Cross 5/9 St., 50-345 Wrocław, Poland; wioletta.galuszka@rckik.wroclaw.pl (W.G.); anna.pecak@rckik.wroclaw.pl (A.P.-S.)

**Keywords:** blood platelets, matrix metalloproteinases, platelet activation, platelet transfusion

## Abstract

The reasons for unfavorable changes in platelet concentrate (PC) quality during storage are not fully understood yet. We aimed to evaluate whether leukocytes and matrix metalloproteinases (MMPs) lead to a decrease in the quality of PCs and examine whether MMP inhibition will slow down the platelets’ aging. Nine PCs were divided into three parts: (1) leukocyte-depleted (F) PCs, (2) PCs with no additional procedures (NF), and (3) PCs with the addition of an MMP inhibitor—doxycycline (D). Each PC was stored for 144 h, and a sample for testing was separated from each part on the day of preparation and after 24, 48, 72 and 144 h of storage. Blood morphological analysis, platelet aggregation, and the expression of activation markers were evaluated. MMP-2 and MMP-9 concentration, activity, and gene expression were assessed. Platelet aggregation decreased, and platelet activation marker expression increased during the storage. D concentrates showed the lowest level of platelet activation. In turn, leukocyte-depleted PCs showed the highest level of platelet activation in general. MMP-9 platelet activity was higher in leukocyte-containing concentrates at the end of the storage period. We concluded that the filtration process leads to a higher platelet activation level. The presence of doxycycline in PCs reduces the expression of the activation markers as compared to leukocyte-depleted concentrates.

## 1. Introduction

Platelet concentrates (PCs) are components that require special storage conditions and have a short, useful life of five to a maximum of seven days [1,2]. During the storage of PCs, a deterioration in their quality is observed, including a loss of platelet function, which is referred to as platelet storage lesions (PSLs) [3]. Adverse changes affect a platelet’s structure and its function, which directly impacts the effectiveness of transfusion [4]. Along with the development of bacterial detection systems and advances in microbial inactivation, it may soon be possible to extend the shelf life of platelets to the extent that the progressive reduction in platelet functionality in PCs will become a major limiting factor [1,3].

The factors leading to a progressive loss of platelet aggregation capacity and progressive activation remain unclear. PSLs develop during both the preparation and storage of PCs [5]. It has been reported that factors leading to platelet activation and damage include contact with a charged surface, changes in temperature and oxygen availability, and the presence of leukocytes and erythrocytes, cellular debris and enzymes. Researchers have studied the contents of RANTES, TGF-β, INF-γ, IL-1, IL-6, and IL-8 in PCs; the extracellular accumulation of histamine, PAI-1,VEGF and IL-6; and platelet lipid profile changes [5,6,7,8,9,10,11,12,13,14]. Changes in pH and changes in the concentration of lactate dehydrogenase, which is used as a marker of cell damage in cell cultures, were analyzed as potential causes of increased platelet activation [15,16,17,18,19,20]. However, not all mechanisms have been successfully elucidated, and PSLs continue to be one of the main lines of research in transfusion medicine. Giving a comprehensive answer to the cause of these changes remains a challenge, but it offers hope for improving the quality of PCs.

In addition to platelets and plasma, erythrocytes and leukocytes are also present in PCs. Leukocytes contain and release growth factors, cytokines, and extracellular matrix metalloproteinases (MMPs), among others, which can directly affect platelet activity. MMPs play many roles in tissue remodeling and the degradation of various proteins in the extracellular matrix. MMPs participate in cell proliferation, migration, and differentiation, among other activities, and are also involved in cell apoptosis, angiogenesis, tissue repair, and the immune response. Changes in the expression and activity of MMPs occur in many physiological processes, including pregnancy and wound healing, but the involvement of MMPs in pathological processes has also been confirmed, e.g., in cardiovascular diseases, primarily atherosclerosis, and disorders of the musculoskeletal system, including osteoarthritis. MMPs also participate in the process of metastasis and cancer progression [21,22,23,24]. MMPs are also present in platelets and plasma and have been shown to have multidirectional effects on thrombocytes [25]. Platelets are contained and released during the activation of MMP-2, which has a pro-aggregative effect and is of particular importance in platelet activation [26,27,28]. On the other hand, the presence of MMP-9 in platelets remains a contentious issue. It has been suggested that the detection of MMP-9 in platelets is due to their mode of isolation, which determines the extent to which the isolated PLTs are contaminated with leukocytes rich in MMP-9 [29,30,31]. Irrespective of the presence of MMP-9 in platelets, its anti-aggregative effects have been demonstrated [32,33]. The presence and function of MMPs in the context of PSLs are subject to further research.

Undoubtedly, there is a need to identify factors and platelet lesions directly related to storage so that the mechanisms underlying platelet activation can be addressed and progressive PSLs can be minimized [34]. The objective of the present paper was to investigate whether the presence of leukocytes, as well as MMP-2 and MMP-9, and the leukocyte-removal procedure may contribute to the deterioration in the quality of platelet concentrates during storage, and whether they are the cause of changes in platelet reactivity and activation. Changes in the expression of *MMP-2* and *MMP-9* genes in platelets during the storage of PCs were also investigated. Whether the use of the MMP inhibitor doxycycline could lead to slower PSL progression was also assessed. The results of this research may be a first step in further exploring the possibility of extending the storage time of high-quality PCs.

## 2. Materials and Methods

### 2.1. Material

Nine PCs prepared in the Professor Tadeusz Dorobisz Regional Centre for Blood Donation and Haemotherapy in Wrocław constituted the primary research material. The study material was prepared and stored precisely under the same conditions as is routine—in accordance with national procedures and standards. The blood units used to prepare the concentrates were obtained from anonymous donors. Each PC was prepared from five buffy coats suspended in plasma and then divided into three equal parts: non-filtered PC not subjected to any additional procedures (NF); leukocyte-depleted PC using a Teruflex Imugard III filter (Terumo BCT, Tokyo, Japan) (F); and non-filtered PC, with the addition of the doxycycline (doxycycline hydrochloride; cat. D3447, Sigma-Aldrich, Merck, Darmstadt, Germany) dissolved in saline (final concentration 10 µM) (D). Each part was stored simultaneously in breathable containers (Platelet Storage Bag—VSE; 1000 mL (Macopharma, Tourcoing, France)), under continuous agitation, in incubators ensuring a constant temperature of 20–24 °C (Helmer PC3200 incubator with PF84 shakers (Helmer, Noblesville, IN, USA)).

**Figure 1:**
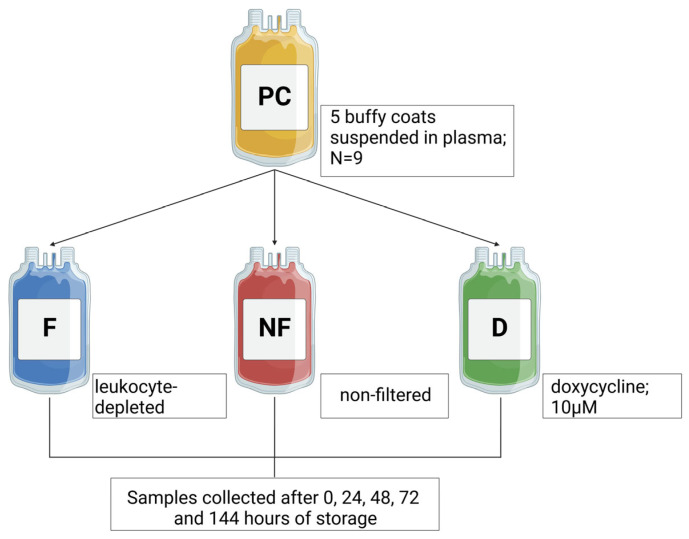
The research scheme of PC preparation and sampling at five points during the storage period. Each PC (*n* = 9) was prepared from five buffy coats suspended in the plasma and then divided into three equal parts: leukocyte-depleted PC (F); non-filtered PC, not subjected to any additional procedures (NF); and non-filtered PC, with the addition of doxycycline (final concentration 10 µM) (D). On the day of preparation (0 h of storage), and after 24 h, 48 h, 72 h and 144 h of PC storage, a test sample was separated from each part.

On the day of preparation (0 h of storage) and then after 24 h, 48 h, 72 h and 144 h of PC storage, a test sample was separated from each part (NF, F, and D). The research scheme is shown in Figure 1.

### 2.2. Methods

Assessments of blood count, platelet aggregation and cytofluorimetric analysis were performed on each sample. Platelets were then isolated from each separated sample, and a medium was prepared. The media was the environment in which platelets were suspended during storage. In the isolated platelets, a relative gene expression was assessed. In the isolated platelets and media, the MMP-2 and MMP-9 activity and concentration were determined.

#### 2.2.1. Blood Count Analysis

Platelet (PLT), leukocyte (WBC) and erythrocyte (RBC) counts, platelet–large-cell ratio (P-LCR), platelet distribution width (PDW) and mean platelet volume (MPV) were assessed using an automated 3-part differential Sysmex KX-21N hematology analyzer (Sysmex, Kobe, Japan). In some cases, the 3-part differential hematology analyzer, especially in samples after 144 h of storage, could not measure PLT or differentiate platelet aggregates from leukocytes. Therefore, some of the samples were subjected to assessment using a Mindray BC-5150 5-part differential hematology analyzer (Mindray, Shenzhen, China).

#### 2.2.2. Aggregation of Platelets

A method using a flat-bottomed 96-well microtiter plate (F98 Maxisorp Nunc-immunoplate, Thermo Fisher Scientific, Waltham, MA, USA) was used to assess aggregation. Aggregation was measured using a SPARK Tecan instrument (Tecan Trading AG, Männedorf, Switzerland). Platelet aggregation was induced using adenosine diphosphate (ADP; final sample concentration 5 µM; cat. AG001K), collagen (COL; final sample concentration 10 µg/mL; cat. AG005K), epinephrine (EPI; final sample concentration 5 µM; cat. AG002K), and arachidonic acid (AA; final sample concentration 750 µM; cat. AG003K) (Hyphen BioMed, Neuville-sur-Oise, France).

Before the measurement of aggregation, platelet counts were assessed in each separated PC sample. All samples were then diluted with Tyrode’s buffer (140 mM NaCl; 2.7 mM KCl; 12 mM NaHCO_3_; 0.4 mM KH_2_PO_4_; 2 mM CaCl_2_; 1 mM MgCl_2_; 0.35% BSA; 0.1% glucose; 20 mM HEPES; pH 7.4), reaching a platelet count of approximately 300 × 10^3^/μL. Subsequently, 10 μL of each agonist (ADP, COL, EPI, AA) and PBS buffer were manually applied to the wells of the plate, always in duplicate for each agonist and PC tested. The plate with the applied agonists was placed in the instrument at 37 °C. Using an automatic dispensing system, 90 μL of the PC was added to each well. Absorbance was measured at 580 nm for approximately 5.5 min—measurements were taken in 40 cycles, with agitation after each cycle (measurement occurred every approximately 8.3 s according to the characteristics of the instrument). After the completion of the aggregation of a given PC and before filling the system with the next sample, the procedure of washing the instrument with the HEPES buffer (140 mM NaCl, 20 mM HEPES, pH 7.4) was performed. The degree of aggregation, expressed in %, was calculated according to the formula:

$$\frac{A580\;PC\;with\;no\;agonist\;-\;A580\;PC\;after\;the\;addition\;of\;the\;agonist}{A580\;PC\;with\;no\;agonist}\times 100\%$$

A580 PC with no agonist—mean absorbance of two measurements using 10 μL of PBS solution instead of the agonist after 40 cycles.

A580 PC after the addition of the agonist—mean absorbance of two measurements using 10 μL of the appropriate agonist after 40 cycles.

#### 2.2.3. Cytofluorimetric Analysis

Cytofluorimetric assessment of platelets was performed using a CyFlow^®^ Space flow cytometer (Sysmex, Kobe, Japan). As markers of platelet activation, the surface expression of P-selectin (CD62P; PE anti-human CD62P Antibody, Clone AK4, cat. 304906, BioLegend, San Diego, CA, USA), activated receptor for fibrinogen—GPIIb/IIIa (PAC-1; FITC anti-human CD41/CD61 Antibody, Clone PAC-1, cat. 362804, BioLegend, San Diego, CA, USA) and the CD63 molecule (PE/Cy7 anti-human CD63 Antibody, Clone H5C6, cat. 353010, BioLegend, San Diego, CA, USA) were assessed. In each sample, 20,000 elements were counted.

#### 2.2.4. Isolation of Platelets

A method for platelet isolation was previously published [35]. The method allows a platelet suspension to be obtained free from ‘contamination’ by other blood cellular elements as well as plasma components. Briefly, a 4-fold diluted PC (with prostacyclin (PGI_2_ sodium salt, cat. P6188, Sigma-Aldrich, Merck, Darmstadt, Germany) supplementation) was layered onto OptiPrep^TM^ with a density gradient of 1.063 g/mL (OptiPrep^TM^ Density Gradient Medium, cat. D1556, Sigma-Aldrich, Merck, Darmstadt, Germany). After centrifugation (300× *g*, 20 min, room temperature), the PGI_2_ was again added to the supernatant, which was followed by further centrifugation (900× *g*, 10 min, room temperature). The platelet pellets were gently resuspended in 1 mL of the HEPES buffer by adding 1 µL of the PGI_2_. The platelet suspension was pipetted into Eppendorf Tubes^®^, the samples were centrifuged (600× *g*, 10 min, room temperature), and the resulting isolated platelet pellets were frozen at −80 °C until the subsequent analyses were performed.

#### 2.2.5. Media Preparation

A part of the sample from each PC was centrifuged to obtain a platelet-free medium. The medium constituted the environment in which the platelets were suspended during their storage. A measure of 3 mL of each PC was centrifuged (900× *g*, 10 min, room temperature), and the supernatant obtained was divided and transferred into Eppendorf Tubes^®^ (volume approximately 1.2 mL). Samples were centrifuged (600× *g*, 10 min, room temperature), and the resulting medium was frozen in portions at −80 °C until the subsequent analyses were performed.

#### 2.2.6. RNA Isolation, Reverse Transcription and Real-Time PCR

Genetic material was obtained from the isolated frozen platelets. For RNA isolation and purification, a two-phase RNA extraction was performed using Trizol^TM^ Reagent (cat. 15596026, Thermo Fisher Scientific, Waltham, MA, USA), chloroform (cat. 602-006-00-4, P.P.H ‘Stanlab’, Lublin, Poland) and isopropanol (cat. 117515002, 2-propanol 99.7% (isopropyl alcohol) p. a., Chempur, Piekary Śląskie, Poland) according to the manufacturer’s instructions for Trizol^TM^ Reagent. RNA concentration was measured using a Nanodrop Lite Spectrophotometer (Thermo Scientific, Waltham, MA, USA).

The RNA obtained through the isolation process was subjected to a reverse transcription (RT) to obtain cDNA. The procedure was carried out using the commercial iScript cDNA Synthesis Kit (cat. 1708890, Bio-Rad, Hercules, CA, USA) according to the procedure of the manufacturer. The resulting cDNA was frozen at −80 °C until the real-time PCR reaction occurred.

The expression of the *MMP-2* and *MMP-9* genes, as well as of the *β-actin* gene as a housekeeping gene [36], was assessed by real-time PCR. A commercially available kit containing all reaction components at the optimal concentrations—the Quantum EvaGreen^®^ PCR Kit (cat. SY570711, Syngen Biotech, Wrocław, Poland)—was used to perform the PCR. The procedure was performed according to the instructions of the manufacturer. Primers with the following sequences were used: β-actin forward 5′-3′: CTCTTCCAGCCTTCCTTCCT; β-actin reverse 5′-3′: AGCACTGTGTTGGCGTACAG; MMP-2 forward 5′-3′: ACAGCAGGTCTCAGCCTCAT; MMP-2 reverse 5′-3′: TGAAGCCAAGCGGTCTAAGT; MMP-9 forward 5′-3′: TTGACAGCGACAAGAAGTGG; and MMP-9 reverse 5′-3′: CCCTCAGTGAAGCGGTACAT. The primers were synthesized by Syngen Biotech, and their sequences were designed using Primer3Plus version 3.3.0 software. The reaction was conducted in a CFX96 Touch Real-Time System thermocycler (Bio-Rad, Hercules, CA, USA). Calculations were performed using the delta-delta Ct (2^−∆∆Ct^).

#### 2.2.7. Gelatin Zymography

The activity of the *MMP-2* and *MMP-9* genes was assessed in the homogenized isolated platelets and in the 25-fold diluted media. Platelet homogenization was performed by adding a homogenization buffer (0.05 M Tris-HCl, 0.15 M NaCl, 0.1% Triton X-100, pH 7.4 with a 1:100 (*v*/*v*) protease inhibitor cocktail) to the pellet obtained during the isolation step (Protease Inhibitors Cocktail, cat. P8340, Sigma-Aldrich, Merck, Darmstadt, Germany). Then, the samples were mechanically homogenized on ice using a Pellet Pestle^®^ Motor (Kimble Kontes, Vineland, NJ, USA). The homogenates were centrifuged, and the supernatants were then used to determine a protein concentration and assess a metalloproteinase activity. Protein concentration was determined by the Bradford method using the Protein Assay Dye Reagent Concentrate (cat. 500-0006, BioRad, Hercules, CA, USA), following the procedure of the manufacturer.

The first stage of zymography was electrophoresis using 7.5% SDS-PAGE with copolymerized gelatin. Electrophoresis was carried out in a Mini-Protean II (BioRad, Hercules, CA, USA). After electrophoresis, the gels were washed three times with 2.5% Triton X-100 to remove SDS and then incubated with an incubation buffer (0.05 M Tris-HCl, pH 7.5 containing 5 mM CaCl_2_, 0.2 M NaCl, 0.05% NaN_3_) at 37 °C for 18 h. Once incubated, the gels were stained with 0.3% Coomassie^®^ brilliant blue R-250 (cat. 04821616-CF, MP Biomedicals, Santa Ana, CA, USA) mixed with 0.2% Coomassie^®^ brilliant blue G-250 (cat. 190343, ICN Biomedicals Inc., Santa Ana, CA, USA) and then decolorized until white bars appeared on a blue background. To measure the activity of the MMPs, zymograms were scanned with a densitometer (GS-800, BioRad, Hercules, CA, USA) and analyzed with the Quantity One software version 4.6.9 (BioRad, Hercules, CA, USA). In each gel, capillary blood was used as a standard, as described by Makowski and Ramsby [37], and the relative MMP activity was expressed in arbitrary units (AUs) per μg of protein in the sample.

#### 2.2.8. Immunoenzymatic Measurement of the MMP-2 and MMP-9 Concentrations

The MMP-2 and MMP-9 concentrations were measured in the isolated, homogenized platelets and in the medium. The MMP-2 and MMP-9 concentrations were measured using the commercial ELISA kits: the Human MMP2 (Matrix Metalloproteinase 2) ELISA Kit (cat. ELK1121) and the Human MMP9 (Matrix Metalloproteinase 9) ELISA Kit (cat. ELK1262) from ELK Biotechnology, USA. The procedure was performed according to the instructions provided by the assay manufacturer. The metalloproteinase concentrations were expressed per mg of protein.

### 2.3. Statistical Analysis

A statistical analysis was performed using Statistica 13.3 (TIBCO Software Inc., Palo Alto, CA, USA). A Friedman repeated measure analysis of variance by ranks (ANOVA) with Dunn’s post hoc test was used to compare a parameter change over time within a single concentrate. The Wilcoxon test for related variables was used to compare the parameter change between the different types of concentrates at a given time point. To investigate the relationship between metalloproteinase activity and a concentration with the platelet activation, the Spearman’s rank correlation coefficients were assessed. A *p*-value < 0.05 was considered to be statistically significant. Results are presented as mean with the standard error of the mean.

## 3. Results

### 3.1. Morphological Changes

**Figure 2:**
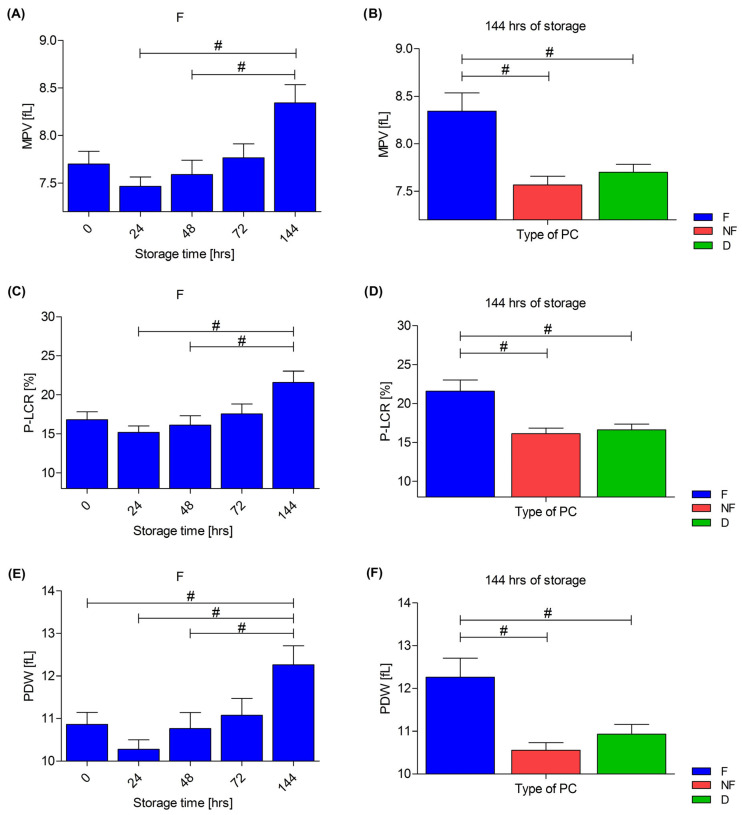
Changes in mean platelet volume—MPV (**A**), platelet–large-cell ratio—P-LCR (**C**) and platelet distribution width—PDW (**E**) in leukocyte-depleted platelet concentrates (PCs) during storage. Comparison of MPV (**B**), P-LCR (**D**) and PDW (**F**) between F—leukocyte-depleted PC, NF—non-filtered PC and D—PC with doxycycline addition after 144 h of storage. The Friedman repeated measure analysis of variance by ranks with Dunn’s post hoc test (**A**,**C**,**E**) and the Wilcoxon test (**B**,**D**,**F**) were used; # *p* < 0.05. Data are presented as mean with SEM. *n* = 9.

During the storage of PCs, the platelet count was stable in every PC type and no differences were found between the three types: F, D and NF. In F, after 144 h of storage, the three-part differential analyzer flagged an ‘AG!’ next to PLT and could not measure PLT or differentiate platelet aggregates and leukocytes. Consequently, changes were observed in the platelet-related parameters—MPV, P-LCR and PDW. Concerning MPV, an increase in this parameter was observed during storage in the F concentrate (*p* < 0.01), as well as differences compared to the D concentrate after 72 h (*p* = 0.027) and after 144 h compared to the D (*p* < 0.01) and to the NF (*p* < 0.01) types (the figure in Section 3.4). An analogous situation occurred for the P-LCR and PDW. Changes in MPV, P-LCR and PDW in leukocyte-depleted PCs are shown in Figure 2.

### 3.2. Aggregation

**Figure 3:**
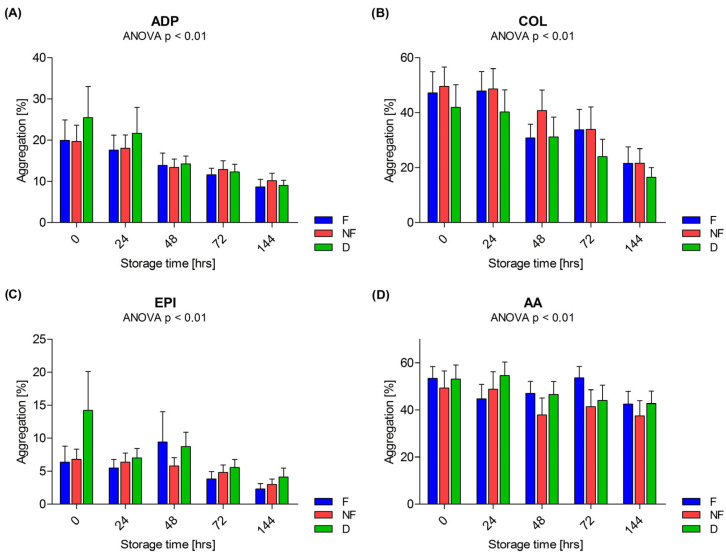
Changes in platelet aggregation induced by adenosine diphosphate (ADP; final sample concentration 5 µM) (**A**), collagen (COL; final sample concentration 10 µg/mL) (**B**), epinephrine (EPI; final sample concentration 5 µM) (**C**) and arachidonic acid (AA; final sample concentration 750 µM) (**D**) in three types of platelet concentrates: F—leukocyte-depleted PC, NF—non-filtered PC, D—PC with doxycycline addition during storage. The Friedman repeated measure analysis of variance by ranks (ANOVA). Data are presented as mean with SEM. *n* = 9.

A reduction in the ability of platelets to aggregate was observed during the storage of the PCs. These changes were observed in all three types of PCs induced by each agonist—ADP, COL, EPI and AA. Changes in platelet aggregation are shown in Figure 3.

### 3.3. Platelet Activation

**Figure 4:**
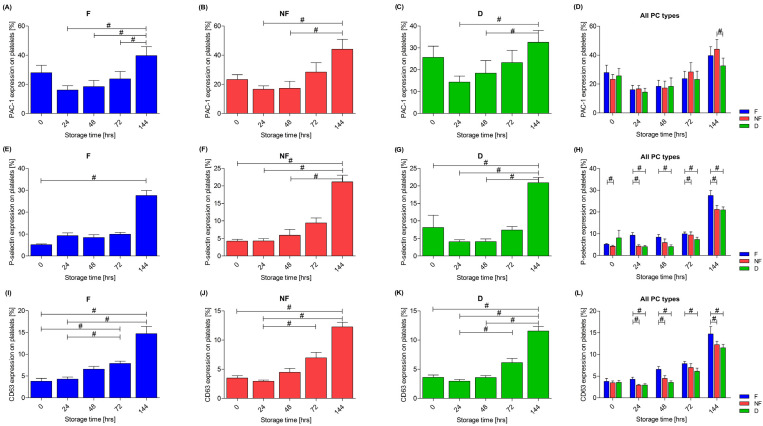
Changes in the surface expression of platelet activation markers—activated receptor for fibrinogen—PAC-1 (**A**–**D**), P-selectin (**E**–**H**) and CD63 (**I**–**L**)—during the storage of leukocyte-depleted (F) PC, non-filtered(NF) PC and PC with doxycycline addition (D). The Friedman repeated measure analysis of variance by ranks with Dunn’s post hoc test (**A**–**C**,**E**–**G**,**I**–**K**) and the Wilcoxon test (**D**,**H**,**L**) were used; # *p* < 0.05. Data are presented as mean with SEM. *n* = 9.

One of the platelet activation markers assessed was the activated receptor for fibrinogen—glycoprotein (GP) IIb/IIIa, which changes a conformation during the platelet activation, resulting in increased binding to the PAC-1 antibody. An increase in the expression of PAC-1 was observed during storage in all three types of PC—the F (*p* < 0.01), the NF (*p* < 0.01) and the D (*p* < 0.01) PCs. A trend towards less activation of this receptor at the end of storage in the PC with doxycycline was also observed, which was confirmed after 144 h of storage, when the expression of PAC-1 was significantly lower in the D concentrate compared to the NF one (*p* = 0.02), as shown in Figure 4A–D.

The second activation marker assessed was the cell adhesion molecule stored in the α granules of platelets, P-selectin. An increase in expression was observed in all three types of PCs during the storage: the F (*p* < 0.01), the NF (*p* < 0.01) and the D (*p* < 0.01) PCs—Figure 4E–G. On the day of preparation (*p* = 0.02), after 24 h (*p* < 0.01) and after 144 h (*p* = 0.015) of storage, the expression of P-selectin was significantly higher in the F PC compared to the NF PC. Moreover, comparing the F concentrate with the D concentrate after 24 h of storage (*p* < 0.01), at all subsequent time points–48 h (*p* = 0.011), 72 h (*p* < 0.01) and 144 h (*p* = 0.01)—the expression of this marker was higher in the F concentrate. At 72 h of storage, a higher expression of P-selectin was also present in the NF PC compared to the D PC (*p* = 0.02), whereas already after 48 h, a pronounced trend towards a lower expression in the D concentrate can be observed. A comparison of the expression of P-selectin across all types of PCs is shown in Figure 4H.

An increase in the expression of CD63 was found in all three types of the PCs during storage: in the F PC (*p* < 0.01), the NF PC (*p* < 0.01) and the D PC (*p* < 0.01)—Figure 4I–K. Differences were observed between the 0–24 h period and after 144 h of storage. Furthermore, only the PC with doxycycline in the post hoc test showed a difference between 48 h and 144 h of storage, at which point the expression of CD63 in the D concentrate still remained relatively low. Following 24 h (*p* = 0.015) and after 48 h (*p* < 0.01) of storage, the expression of CD63 was significantly higher in the leukocyte-depleted PC compared to the NF PC. When comparing the NF concentrate with the D one, a higher expression of CD63 was found after 144 h of storage in the NF PC (*p* = 0.028). A comparison of the expression of CD63 across all the types of PCs is shown in the Figure 4L.

### 3.4. Extracellular Matrix Metalloproteinases

Analysis of a zymogram showed that the highest activity of enzymes with a molecular weight of 92 kDa corresponded to the proMMP-9 form, and a molecular weight of 72 kDa corresponded to the proMMP-2 form, which are referred to as MMP-9 and MMP-2, respectively, throughout the rest of this paper.

**Figure 5:**
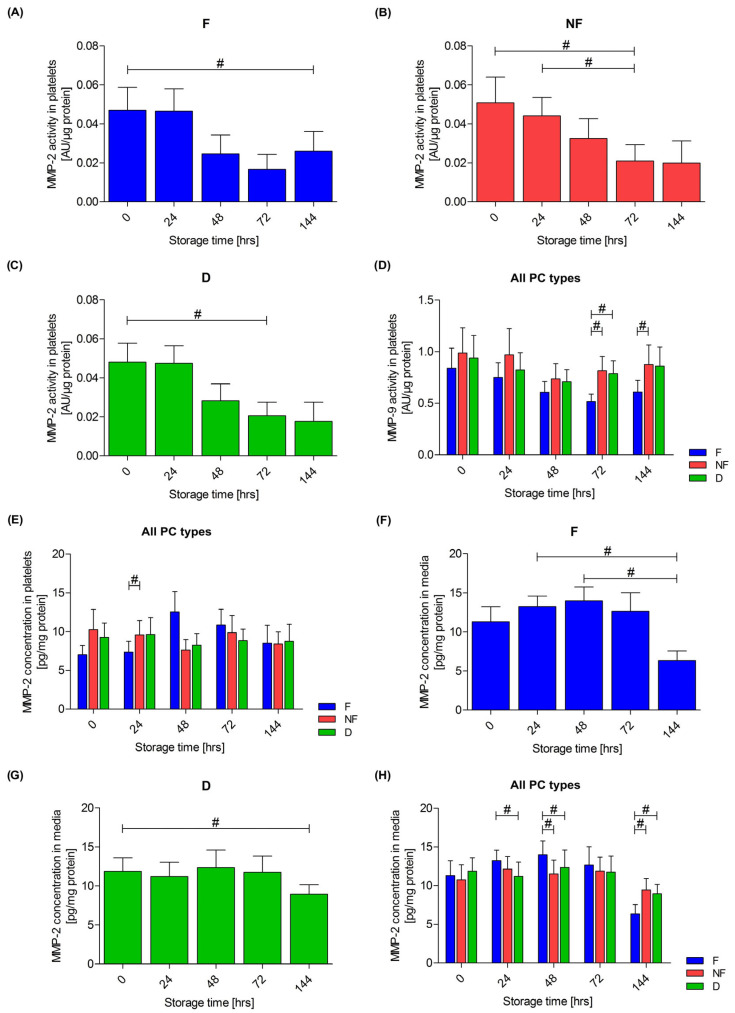
Changes in MMP-2 activity in platelets during storage of leukocyte-depleted (F) PC (**A**), non-filtered (NF) (**B**) and PC with doxycycline addition (D) (**C**). Comparison of MMP-9 activity in platelets in all types of platelet concentrates (PCs) (**D**). Comparison of MMP-2 concentration in platelets in all types of platelet concentrates (**E**). Changes in MMP-2 concentration in media during the storage of leukocyte-depleted (F) PCs (**F**) and PCs with doxycycline addition (D) (**G**). Comparison of MMP-2 concentration in media in all types of platelets concentrates (**H**). The Friedman repeated measure analysis of variance by ranks with Dunn’s post hoc test (**A**–**C**,**F**,**G**) and the Wilcoxon test (**D**,**E**,**H**) were used; # *p* < 0.05. Data are presented as mean with SEM. *n* = 9.

In the isolated platelets, a reduction in the activity of MMP-2 was observed during the storage of all three types of PCs: the F PC (*p* < 0.01), the NF PC (*p* = 0.01) and the D PC (*p* < 0.01), as shown in Figure 5A–C. No differences were observed between the different types of PCs during storage. Going further, no changes in MMP-2 activity were observed in the medium during storage, and there were no differences between the PCs.

No change in the MMP-9 activity in platelets was observed during the storage of any PC. However, a difference in activity was found between the F and NF PCs after 72 h of storage (*p* = 0.01) and after 144 h of storage (*p* = 0.038), as well as between the F and D concentrates after 72 h of storage (*p* = 0.01), as shown in Figure 5D. Whenever the PC was filtered, the activity of MMP-9 was lower. No change in the MMP-9 activity was observed during the storage of PCs for the medium, and no differences were found between the different concentrate types.

The concentration of MMP-2 in platelets did not differ between days of storage in any type of PC. Changes and differences in MMP-2 concentration in platelets are shown in Figure 5E. A difference was observed between the F and NF PCs after 24 h of storage (*p* = 0.038). Changes in the concentration of MMP-2 in the medium during the storage of PCs were found in the F (*p* < 0.01) and D (*p* = 0.02) PCs, as shown in Figure 5F–G, while no difference was observed in the NF PC (*p* = 0.06). In the NF and D PCs, the higher concentrations of MMP-2 in the medium were observed compared to the F PC after 144 h of storage (*p* < 0.01). However, the contrary observation was after 24 and 48 h of storage, as shown in the Figure 5H. No differences in the concentration of MMP-9 in platelets were observed during the storage of the PCs. The only observed difference in the concentration of MMP-9 in the medium was during the storage of the F concentrate. No differences were observed between the respective types of PCs.

**Figure 6:**
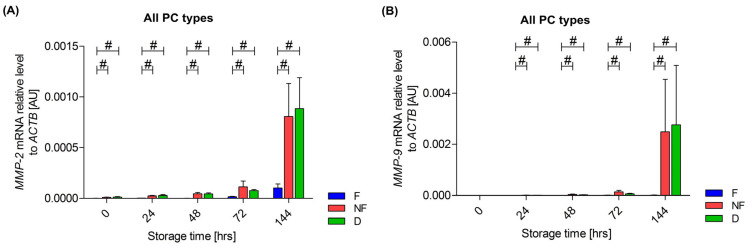
Differences in *MMP-2* (**A**) and *MMP-9* (**B**) gene expression in platelets between the three platelet concentrates (PCs) types: leukocyte-depleted (F) PC, non-filtered (NF) PC and PC with the addition of doxycycline (D) on the day of preparation (0 h of the storage) and after 24 h, 48 h, 72 h and 144 h of storage. The Wilcoxon test was used, # *p* < 0.05. Data are presented as mean with SEM. *n* = 6.

An increase in the expression of MMP-2 mRNA was observed during the storage of all three types of PCs: the F (*p* < 0.01), NF (*p* < 0.01) and D (*p* < 0.01) PCs. The differences between the individual types of PCs were observed at all time points. The difference was always between the NF and the F PCs (*p* = 0.02) and the D and the F PCs (*p* = 0.02), and the expression was always lowest in the F PC, as shown in Figure 6A. An increase in the expression of MMP-9 mRNA was also observed during the storage of two types of PCs—the NF (*p* < 0.01) and D (*p* < 0.01) PCs, while no such changes were observed in the F PC (*p* = 0.38). The differences between individual PC types were observed between the F and NF PCs, and the F and D PCs from 24 h of storage (24 h, *p* = 0.02 and *p* = 0.046; 48 h, *p* = 0.02 and *p* = 0.02; 72 h, *p* = 0.02 and *p* = 0.046; 144 h, *p* = 0.02 and *p* = 0.02, respectively). The expression was always the lowest in the F concentrate. A comparison of the changes over time between the three types of PCs is shown in Figure 6B.

### 3.5. Relationship of Metalloproteinases with Platelet Activation and Leukocyte Count

**Figure 7:**
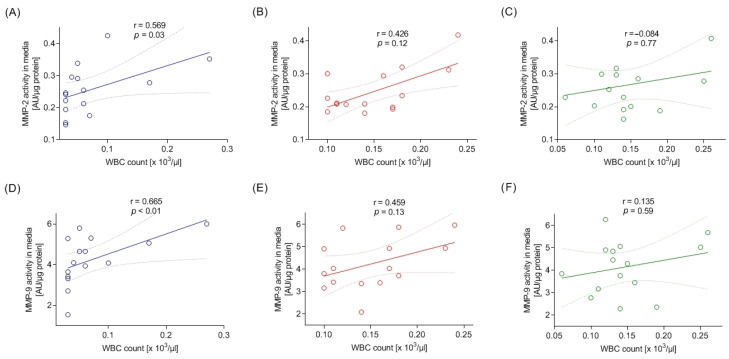
Spearman’s rank correlation coefficients for MMP-2 and MMP-9 activity in media and leukocyte (WBC) count (5-diff measurement) in three types of platelet concentrates: leukocyte-depleted (**A**,**D**), non-filtered (**B**,**E**) and with the addition of doxycycline (**C**,**F**); *n* = 3. Dots represents data, two confidence bands (dotted lines) surrounding the best-fit line (solid line) define the 95% confidence interval of the best-fit line.

A moderately strong correlation between the activity of MMP-2 and MMP-9 in the media in which doxycycline was not used and the number of leukocytes was found (due to the higher accuracy of the determinations, the measurement from the five-part differential analyzer was used for calculations). In some cases, a statistical significance was not obtained, which may have been due to the smaller number of samples subjected to a five-part differential analysis. Figure 7 shows Spearman’s rank correlation coefficients between the leukocyte count and the MMP activity.

There was no unambiguous relationship between the MMP activity in platelets and activation markers. A summary of the Spearman’s rank correlation coefficients for the activity of the MMPs in platelets and the expression of activation markers is presented collectively in Table 1. When analyzing the correlation of MMP-2 and MMP-9 concentrations in the media with the PAC-1 activation marker, a moderate positive correlation was observed in the concentrates containing leukocytes (NF and D). In the non-filtered PCs, the correlation coefficient between the MMP-2 and the PAC-1 was r = 0.285 (*p* = 0.058), and between MMP-9 and PAC-1 it was r = 0.330 (*p* = 0.027). In the doxycycline concentrate, the correlation coefficients between MMP-2 and PAC-1 and MMP-9 and PAC-1 were r = 0.408 (*p* < 0.01) and r = 0.343 (*p* = 0.021), respectively.

## 4. Discussion

During the storage of the PCs, adverse changes in platelets—PSLs—occur [38]. The reasons why platelets lose their ability to aggregate and undergo intensified activation during storage remain unclarified [5,14]. Platelets, plasma and leukocytes present in the PC contain and release, inter alia, extracellular matrix metalloproteinases that affect the activity of platelets [25]. The aim of the study was to verify whether the presence of leukocytes and MMPs or employing a leukocyte-removal procedure can contribute to PSL, and whether the use of the MMP inhibitor doxycycline may lead to a slower progression of the PSL process.

No changes regarding the platelet count were observed during the storage of the PCs. The platelet count was also not affected by the type of concentrate (F, NF or D). In most of the available data, no changes in the platelet count were observed during the storage, irrespective of the method of preparation [4,16,17,39,40,41,42,43], but there are also reports indicating a decrease in the number of thrombocytes during the storage of PCs [44,45]. In some papers, the MPV did not change over time [5,40,41], which is not consistent with the results obtained by us. An increase in MPV was observed during the storage in the leukocyte-depleted concentrates, as well as a higher MPV in the F PC compared to the unfiltered concentrates (NF and D). Importantly, the occurrence of the “AG!” flag when measuring the platelet count after 144 h in F concentrate means that the analyzer was unable to determine the boundary separating platelet aggregates from leukocytes. This undoubtedly affected the changes observed in the parameters related to platelets—MPV, P-LCR and the PDW. It is worth noting that those indices increase in the presence of the aggregates of platelets. Thus, the results suggest that the filtration process may have contributed to the increased formation of the platelet aggregates during the last days of PC storage. Seghatchian found no significant difference in MPV, PDW or P-LCR immediately after the preparation of the PCs but found slightly higher values of P-LCR and PDW during storage, suggesting the formation of aggregates [39]. On the other hand, Amorini et al. showed significantly increased MPV after five days of PC storage, and a gradual increase was observed by the eighth day of the storage. All concentrates were prepared from a buffy coat and were not subjected to filtration [45]. The present results indicate that the filtration process itself contributes to the formation of aggregates in the PCs during storage, but this may not be the only cause.

One of the more frequently discussed problems with PSL is the reduction in the ability of platelets to aggregate, regardless of the agonists used. It was observed that after 5 days of PC storage, the capability of thrombocytes to degranulate decreased [4]. It was found that the aggregation induced by ADP decreased linearly during PC storage for 10 days [40]. Similar reductions in aggregation were observed with AA, thrombin receptor activator peptide (TRAP), ristocetin, collagen, ADP and EPI [17,19,46]. In the present research, we also found a reduction in the aggregation capability of platelets induced by all agonists assessed (ADP, COL, EPI and AA). The preparation procedure had no effect on platelet aggregation. The greatest reduction was observed when collagen was applied, with an average reduction of 25.59% for the F PC, 27.91% for the NF PC, and 25.43% for the D PC.

One of the reasons for the loss of the capability of platelets to aggregate may be their increasing activation during storage. An increase in the spontaneous (agonist-free) expression of P-selectin on the platelet surface has been observed regardless of the filtration technique of PCs, as well as in the case of the non-filtered concentrates [5,16,17,19,40,46,47,48], which is consistent with the results obtained in this research. In all types of PCs, an increase in the expression of P-selectin was observed. Remarkably, it was also observed that for the PCs that were not subjected to the filtration process (NF and D), the expression of P-selectin was significantly different after 0 h, 24 h and 48 h compared to after 144 h of the storage—in the early days, it remained at a relatively low, constant level (in the D PC there is a slight downward trend). No such observation was found in the leukocyte-depleted PC, and an upward trend was observed after 24 h. It was also ascertained that the leukocyte-depleted PCs were generally characterized by a higher expression level of CD62P compared to the non-filtered concentrates (NF and D). This suggests that the filtration process itself may lead to increased activation of platelets. The second marker indicating the progressing activation process is the activated form of GPIIb/IIIa. In the present research, an increase in the binding of PAC-1 was observed during the storage of the PCs. There was also a tendency for a higher expression of PAC-1 on the day of preparation of the concentrates (0 h of the storage) compared to the next day (24 h of the storage), at which point platelets are subject to “silencing”. This suggests that the preparation itself has a stimulatory effect on platelets. Other conclusions were reached by Kicken et al. and Plaza et al., who showed that the storage of a PC had no significant effect on the spontaneous activation of the GPIIb/IIIa receptor [40,46]. However, the stimulation of platelets with ADP on the first day of the storage induced a conformational change in the receptor in 56.4 ± 14.4% of platelets, as assessed by the binding of PAC-1. After five days of storage, a reduction in this response was observed, and after 10 days, only 15.9 ± 5.1% of ADP-activated platelets bound PAC-1 [46]. The third platelet activation marker was the CD63 molecule, a lysosome-related protein. As with PAC-1 and P-selectin, an increase in the expression of CD63 was found in all types of PCs, which is consistent with other studies [46,49]. Interestingly, in the case of PCs not subjected to filtration (NF and D), a slight decreasing trend was observed during the first 48 h of storage. In addition, it was observed that the leukocyte-depleted PC generally shows a higher expression of the CD63 molecule compared to the unfiltered components (NF and D). This confirms again that the filtration procedure of the PC itself can lead to an over-stimulation of platelets. On the other hand, Vucic et al. found an increase in CD63 expression during the storage PCs but a lower expression of this molecule in the leukocyte-depleted concentrates [49]. A simultaneous assessment of different markers of platelet activation can be an effective tool to assess the quality of the PC during storage. Unfortunately, there is also no established benchmark that can be used to convert the measured loss of PLT function in vitro into a corresponding quality evaluation of platelets in vivo. Given the above, it appears that a better understanding of the activation of platelets during storage may provide new insights into the development of novel approaches to the preparation of platelet concentrates [43,49]. Moreover, researchers suggest that platelet subpopulations, distinguished based on the expression of markers of activation, apoptosis (annexin V) and released microparticles, also show different aggregation abilities. Platelet subpopulations may also differ in MPV, and the centrifugation of concentrates during preparation may result in the loss of very large or very small platelets. It appears that the isolation of functionally distinct platelet subpopulations may be desirable when preparing platelet concentrates. The percentage of a given type of platelet (procoagulant or apoptotic) may also be influenced by the method of storage, e.g., cryopreservation [17,50,51,52].

Studies on protease inhibitors have revealed that metalloproteinases are mainly responsible for proteolytic changes during the storage of PCs. Incubation of human platelets for up to seven days with marimastat, a broad-spectrum MMP inhibitor, showed improved platelet response to agonists, supporting the hypothesis that marimastat inhibits platelet activation during storage [53]. The only inhibitor of MMPs approved by the FDA is doxycycline. Moreover, doxycycline was confirmed not to hyperactivate platelets [54]. Thus, in the present research, an attempt was made to reduce the activity of MMPs in the PCs to slow down the activation of platelets and thereby improve the quality of the PCs by adding doxycycline. After 144 h of storage, lower concentrations of MMP-2 were found in the leukocyte-depleted PC. This observation might suggest that the progressive breakdown of leukocytes (as indicated by the results from the five-part differentiation analyzer) contributes to an increase in the concentrations of metalloproteinases. However, this increase was not confirmed for MMP-9. It was demonstrated that the activity of MMP-2 in platelets was reduced during their storage, irrespective of platelet preparation. MMP-2 is involved in the adhesion and aggregation of platelets, which also decreased during storage. The activity of MMP-9 in platelets did not change significantly during the storage of the different types of the PCs, but in the leukocyte-depleted concentrate, the activity was lower as compared to the PCs containing leukocytes (NF and D). Despite the inability to demonstrate reduced gelatinolytic activity in PCs with the addition of doxycycline, a moderately strong correlation was found between the activity of MMP-2 and MMP-9 and the number of leukocytes only in the medium of the PCs without the addition of doxycycline (F and NF). Going further, no clear correlation was observed between the activation of platelets and the activity of metalloproteinases. However, it was observed that in leukocyte-containing concentrate media, MMP-2 and MMP-9 concentrations showed a moderate correlation with the expression of the activated GPIIb/IIIa. After 144 h of storage, the activation of GPIIb/IIIa was lower in the D concentrate compared to the NF one. Also, the expression of P-selectin in the D PC was lower (at 24 h of storage) compared to the other two types of PC. A similar observation was made in the case of CD63, where from 24 h of storage, the doxycycline-enhanced concentrate also had the lowest expression of this molecule. The results suggest that the addition of doxycycline may have a beneficial effect on the progressive activation of platelets during storage. Undoubtedly, research is still needed on whether preserving human platelets with an MMP inhibitor will provide benefits after transfusion. It has been shown that MMP inhibition (by the addition of GM6001) significantly improved the recovery of mouse platelets after transfusion. The inhibition of metalloproteinases also prevented the proteolysis of GPIbα on the platelet storage lesions, thereby improving the hemostatic function of thrombocytes in vivo [55].

Mature platelets receive many of the components necessary for gene regulation from megakaryocytes (mRNA, pre-miRNA). During storage, platelets continue to translate mRNA into proteins and process pre-miRNAs into mature miRNAs. There is a strong correlation between the platelet transcriptome and their proteomic profile, which confirms the existence of de novo platelet translation capacity. This suggests the possibility of a post-transcriptional regulation of gene expression in platelets under the storage conditions [56]. In this study, the expression of the MMP-2 gene was observed to increase during storage in all types of PCs, while the expression of the MMP-9 gene increased only in the preparations containing leukocytes, the breakdown of which can be a source of mRNA for platelets. During storage, differences in the expression of both genes were found in different types of PCs—in F concentrates, this expression was at the lowest level. This observation may support the hypothesis that since platelets sequester RNA [57,58], it is possible that when stored in an environment where it is present, they have the ability to ‘absorb’ it. The hypothesis of the capability of platelets to absorb substances from the external environment is also supported by the fact that the concentration of this gelatinase in platelets increased with the decrease in the concentration of MMP-2 in the media.

The limitation of the study is the lack of assessment of pH, LDH or glucose changes in concentrates during their storage. It should be noted that the concentrates were prepared and stored in accordance with national procedures, and the Quality Assurance Department at the center is responsible for their appropriate quality. Nevertheless, LDH assay or blood gas analysis parameters would be valuable additional information regarding platelet viability.

To sum up, it has been shown that platelets do not remain indifferent to the process of leukocyte removal, as it causes their activation. On the other hand, one of the sources of MMPs in PCs may be leukocytes, and inhibiting the activity of these enzymes seems to be a promising tool for improving platelet function during their storage. Of course, whether storing human platelets with an MMP inhibitor will provide benefits after transfusion remains to be investigated. The results of our research may be the next step in further searching for possibilities for extending the storage time of high-quality platelet concentrates and modifying the process of their preparation.

## 5. Conclusions

This study confirms the involvement of metalloproteinases in the PSL process. It cannot be concluded that MMPs are the main cause of quality deterioration during storage; however, the presence of the MMP inhibitor doxycycline may have a beneficial effect on the progressive activation of platelets. The results of our research may be the next step in further searching for possibilities for extending the storage time of high-quality platelet concentrates and modifying the process of their preparation.

## Figures and Tables

**Table 1:** Spearman’s rank correlation coefficients for MMP-2 and MMP-9 activity in isolated platelets and the expression of surface markers of platelet activation in three types of concentrates (PC): leukocyte-depleted (F), non-filtered (NF) and with the addition of doxycycline (D).

	Correlation between MMP Activity in Isolated Platelets and Activation Marker		
PC Type	PAC-1 Expression	P-Selectin Expression	CD63 Expression
	***MMP-2***		
F	r = −0.218; *p* = 0.15	r = −0.270; *p* = 0.08	r = −0.269; *p* = 0.07
NF	r = −0.383; *p* < 0.01	r = −0.330; *p* = 0.03	r = −0.336; *p* = 0.02
D	r = −0.239; *p* = 0.11	r = 0.013; *p* = 0.93	r = −0.060; *p* = 0.69
	***MMP-9***		
F	r = 0.060; *p* = 0.70	r = 0.026; *p* = 0.87	r = −0.038; *p* = 0.80
NF	r = −0.059; *p* = 0.70	r = 0.138; *p* = 0.37	r = 0.033; *p* = 0.83
D	r = 0.046; *p* = 0.77	r = 0.173; *p* = 0.26	r = 0.225; *p* = 0.14

## Data Availability

Data available on request.

## References

[1] Naghadeh H.T., A Badlou B., Ferizhandy A.S., Mohammadreza T.S., Shahram V. (2013). Six hours of resting platelet concentrates stored at 22–24 °C for 48 hours in permeable bags preserved pH, swirling and lactate dehydrogenase better and caused less platelet activation. Blood Transfus..

[2] Thomas S. (2016). Platelets: Handle with care. Transfus. Med..

[3] Thon J.N., Schubert P., Devine D.V. (2008). Platelet Storage Lesion: A New Understanding from a Proteomic Perspective. Transfus. Med. Rev..

[4] Black A., Orsó E., Kelsch R., Pereira M., Kamhieh-Milz J., Salama A., Fischer M.B., Meyer E., Frey B.M., Schmitz G. (2017). Analysis of platelet-derived extracellular vesicles in plateletpheresis concentrates: A multicenter study. Transfusion.

[5] Costa E.J., Guimarães T.M.P.D., de Almeida N.C., de Toledo V.d.P.C.P. (2012). Comparison of cytokine levels and metabolic parameters of stored platelet concentrates of the Fundação Hemominas, Belo Horizonte. Brazil Rev. Bras. Hematol. E Hemoter..

[6] Egidi M.G., D’Alessandro A., Mandarello G., Zolla L. (2010). Troubleshooting in platelet storage temperature and new perspectives through proteomics. Blood Transfus..

[7] Estebanell E., Diaz-Ricart M., Lozano M., Mazzara R., Escolar G., Ordinas A. (1998). Cytoskeletal reorganization after preparation of platelet concentrates, using the buffy coat method, and during their storage. Haematologica.

[8] Hoareau G.L., Jandrey K.E., Burges J., Bremer D., Tablin F. (2014). Comparison of the platelet-rich plasma and buffy coat protocols for preparation of canine platelet concentrates. Vet. Clin. Pathol..

[9] Edvardsen L., Taaning E., Dreier B., Christensen L.D., Mynster T., Nielsen H.J. (2001). Extracellular accumulation of bioactive sub-stances during preparation and storage of various platelet concentrates. Am. J. Hematol..

[10] Kaur D., Sharma R.R., Marwaha N. (2015). Defining an appropriate leucoreduction strategy by serial assessment of cytokine levels in platelet concentrates prepared by different methods. Asian J. Transfus. Sci..

[11] Ferrer F., Rivera J., Lozano M.L., Corral J., Garcia V.V. (2001). Effect of cold-storage on the accumulation of bioreactive substances in platelet concentrates treated with second messenger effectors. Haematologica.

[12] Kanter J., Khan S.Y., Kelher M., Gore L., Silliman C.C. (2008). Oncogenic and angiogenic growth factors accumulate during routine storage of apheresis platelet concentrates. Clin. Cancer Res..

[13] Cognasse F., Boussoulade F., Chavarin P., Acquart S., Fabrigli P., Lamy B., Garraud O. (2006). Release of potential immunomodulatory factors during platelet storage. Transfusion.

[14] Allan H.E., Vadgama A., Armstrong P.C., Warner T.D. (2023). What can we learn from senescent platelets, their transcriptomes and proteomes?. Platelets.

[15] Ghezelbash B., Kafibad S.A., Hojjati M.T., Hamidpoor M., Vaeli S., Tabtabae M.R., Gharehbaghian A. (2015). In Vitro Assessment of Platelet Lesions during 5-day Storage in Iranian Blood Transfusion Organization (IBTO) centers. Arch. Iran. Med..

[16] Perrotta P.L., Perrotta C.L., Snyder E.L. (2003). Apoptotic activity in stored human platelets. Transfusion.

[17] Vučetić D., Ilić V., Vojvodić D., Subota V., Todorović M., Balint B. (2018). Flow cytometry analysis of platelet populations: Usefulness for monitoring the storage lesion in pooled buffy-coat platelet concentrates. Blood Transfus..

[18] Ahmed A.S., Leheta O., Younes S. (2010). In vitro assessment of platelet storage lesion in leukoreduced random donor platelet concentrates. Blood Transfus..

[19] Abonnenc M., Sonego G., Kaiser-Guignard J., Crettaz D., Prudent M., Tissot J.-D., Lion N. (2015). In vitro evaluation of pathogen-inactivated buffy coat-derived platelet concentrates during storage: Psoralen-based photochemical treatment step-by-step. Blood Transfus..

[20] Jurisic V., Radenkovic S., Konjevic G. (2015). The Actual Role of LDH as Tumor Marker, Biochemical and Clinical Aspects. Adv. Exp. Med. Biol..

[21] Cui N., Hu M., Khalil R.A. (2017). Biochemical and Biological Attributes of Matrix Metallo-proteinases. Prog. Mol. Biol. Transl. Sci..

[22] Serra R. (2020). Matrix Metalloproteinases in Health and Disease. Biomolecules.

[23] Kapoor C., Vaidya S., Wadhwan V., Hitesh, Kaur G., Pathak A. (2016). Seesaw of matrix metalloproteinases (MMPs). J. Cancer Res. Ther..

[24] Radenkovic S., Konjevic G., Jurisic V., Karadzic K., Nikitovic M., Gopcevic K. (2014). Values of MMP-2 and MMP-9 in tumor tissue of basal-like breast cancer patients. Cell Biochem. Biophys..

[25] Seizer P., May A.E. (2013). Platelets and matrix metalloproteinases. Thromb. Haemost..

[26] Falcinelli E., Guglielmini G., Torti M., Gresele P. (2005). Intraplatelet signaling mechanisms of the priming effect of matrix metalloproteinase-2 on platelet aggregation. J. Thromb. Haemost..

[27] Momi S., Falcinelli E., Giannini S., Ruggeri L., Cecchetti L., Corazzi T., Libert C., Gresele P. (2009). Loss of matrix metalloproteinase 2 in platelets reduces arterial thrombosis in vivo. J. Exp. Med..

[28] Sebastiano M., Momi S., Falcinelli E., Bury L., Hoylaerts M.F., Gresele P. (2017). A novel mechanism regulating human platelet activation by MMP-2–mediated PAR1 biased signaling. Blood.

[29] Kälvegren H., Jönsson S., Jonasson L. (2011). Release of matrix metalloproteinases-1 and -2, but not -9, from activated platelets measured by enzyme-linked immunosorbent assay. Platelets.

[30] Wrzyszcz A., Woźniak M. (2012). On the origin of matrix metalloproteinase-2 and -9 in blood platelets. Platelets.

[31] Mannello F., Medda V. (2011). Differential expression of MMP-2 and MMP-9 activity in megakaryocytes and platelets. Blood.

[32] Fernandez-Patron C., Martinez-Cuesta M.A., Salas E., Sawicki G., Wozniak M., Radomski M.W., Davidge S.T. (1999). Differential Regulation of Platelet Aggregation by Matrix Metalloproteinases-9 and -2. Thromb. Haemost..

[33] Sheu J.R., Fong T.H., Liu C.M., Shen M.Y., Chen T.L., Chang Y., Lu M.S., Hsiao G. (2004). Expression of matrix metalloproteinase-9 in human platelets: Regulation of platelet activation in in vitro and in vivo studies. Br. J. Pharmacol..

[34] Ng M.S.Y., Tung J.-P., Fraser J.F. (2018). Platelet Storage Lesions: What More Do We Know Now?. Transfus. Med. Rev..

[35] Wrzyszcz A., Urbaniak J., Sapa A., Woźniak M. (2017). An efficient method for isolation of representative and contamination-free population of blood platelets for proteomic studies. Platelets.

[36] Zsóri K., Muszbek L., Csiki Z., Shemirani A. (2013). Validation of reference genes for the determination of platelet transcript level in healthy individuals and in patients with the history of myocardial infarction. Int. J. Mol. Sci..

[37] Makowski G.S., Ramsby M.L. (1996). Calibrating Gelatin Zymograms with Human Gelatinase Standards. Anal. Biochem..

[38] Seghatchian J., Krailadsiri P. (1997). The Platelet Storage Lesion. Transfus. Med. Rev..

[39] Seghatchian J. (2006). A new platelet storage lesion index based on paired samples, without and with EDTA and cell counting: Comparison of three types of leukoreduced preparations. Transfus. Apher. Sci..

[40] Kicken C.H., Roest M., Henskens Y.M.C., de Laat B., Huskens D. (2017). Application of an optimized flow cytometry-based quantification of Platelet Activation (PACT): Monitoring platelet activation in platelet concentrates. PLoS ONE.

[41] Slichter S.J., Bolgiano D., Corson J., Jones M.K., Christoffel T., Bailey S.L., Pellham E. (2014). Extended storage of buffy coat platelet concentrates in plasma or a platelet additive solution. Transfusion.

[42] Krailadsiri P., Seghatchian J., Williamson L.M. (2001). Platelet storage lesion of WBC-reduced, pooled, buffy coat-derived platelet concentrates prepared in three in-process filter/storage bag combinations. Transfusion.

[43] Black A., Pienimaeki-Roemer A., Kenyon O., Orsó E., Schmitz G. (2015). Platelet-derived extracellular vesicles in plateletpheresis concentrates as a quality control approach. Transfusion.

[44] Amorini A.M., Tuttobene M., Tamasello F.M., Biazzo F. (2013). Glucose ameliorates the metabolic profile and mitochondrial function of platelet concentrates during storage in autologous plasma. Blood Transfus..

[45] Amorini A.M., Tuttobene M., Lazzarino G., Denti G. (2007). Evaluation of biochemical parameters in platelet concentrates stored in glucose solution. Blood Transfus..

[46] Plaza E.M., Lozano M.L., Guiu I.S., Egea J.M., Vicente V., De Terán L.C., Rivera J. (2012). Evaluation of platelet function during extended storage in additive solution, prepared in a new container that allows manual buffy-coat platelet pooling and leucoreduction in the same system. Blood Transfus..

[47] Canault M., Duerschmied D., Brill A., Stefanini L., Schatzberg D., Cifuni S.M., Bergmeier W., Wagner D.D. (2010). p38 mitogen-activated protein kinase activation during platelet storage: Consequences for platelet recovery and hemostatic function in vivo. Blood.

[48] Seghatchian J. (2006). Platelet storage lesion: An update on the impact of various leukoreduction processes on the biological response modifiers. Transfus. Apher. Sci..

[49] Vucic M., Stanojkovic Z., Antic A., Vucic J., Pavlovic V. (2018). Evaluation of platelet activation in leukocyte-depleted platelet concentrates during storage. Bosn. J. Basic Med. Sci..

[50] Johnson L., Lei P., Waters L., Padula M.P., Marks D.C. (2023). Identification of platelet subpopulations in cryopreserved platelet components using multi-colour imaging flow cytometry. Sci. Rep..

[51] Lesyk G., Jurasz P. (2019). Advances in Platelet Subpopulation Research. Front. Cardiovasc. Med..

[52] Handtke S., Steil L., Greinacher A., Thiele T. (2018). Toward the Relevance of Platelet Subpopulations for Transfusion Medicine. Front. Med..

[53] Prudova A., Serrano K., Eckhard U., Fortelny N., Devine D.V., Overall C.M. (2014). TAILS N-terminomics of human platelets reveals pervasive metalloproteinase-dependent proteolytic processing in storage. Blood.

[54] Bartoli C.R., Kang J., Restle D.J., Zhang D.M., Shabahang C., Acker M.A., Atluri P. (2015). Inhibition of ADAMTS-13 by Doxycycline Reduces von Willebrand Factor Degradation During Supraphysiological Shear Stress: Therapeutic Implications for Left Ventricular Assist Device-Associated Bleeding. JACC Heart Fail..

[55] Bergmeier W., Burger P.C., Piffath C.L., Hoffmeister K.M., Hartwig J.H., Nieswandt B., Wagner D.D. (2003). Metalloproteinase inhibitors improve the recovery and hemostatic function of in vitro–aged or–injured mouse platelets. Blood.

[56] Yan Y., Zhang J., Zhang Q., Chen Y., Zhu X., Xia R. (2017). The role of microRNAs in platelet biology during storage. Transfus. Apher. Sci..

[57] Nilsson R.J.A., Balaj L., Hulleman E., Van Rijn S., Pegtel D.M., Walraven M., Widmark A., Gerritsen W.R., Verheul H.M., Vandertop W.P. (2011). Blood platelets contain tumor-derived RNA biomarkers. Blood.

[58] Nilsson R.J.A., Karachaliou N., Berenguer J., Gimenez-Capitan A., Schellen P., Teixido C., Tannous J., Kuiper J.L., Drees E., Grabowska M. (2015). Rearranged EML4-ALK fusion transcripts sequester in circulating blood platelets and enable blood-based crizotinib response monitoring in non-small-cell lung cancer. Oncotarget.

